# Pricing Policies of Green Dual-Channel Supply Chain with Fairness Concerns and Altruistic Preferences Based on Consumers’ Environmental Awareness and Channel Preference

**DOI:** 10.3390/ijerph192013564

**Published:** 2022-10-19

**Authors:** Genhasi Ge, Daoping Wang, Mesumbe Bianca Epede

**Affiliations:** 1 School of Economics and Management, University of Science and Technology Beijing, Beijing 100083, China; 2 School of Statistics and Mathematics, Inner Mongolia University of Finance and Economics, Hohhot 010070, China

**Keywords:** pricing policies, green dual-channel supply chain, fairness concern, altruistic preference, CEA, channel preference

## Abstract

Taking into consideration fairness concerns and altruistic preferences of manufacturers, this paper aims to propose a green dual-channel supply chain that incorporates consumers’ environmental awareness (CEA) and channel preference. The purpose of this work is to explore and further compare the optimal outcomes in a green dual-channel supply chain in three scenarios, which are the fairness-neutrality scenario (Model *N*), the manufacturer is concerned with fairness scenario (Model *F*), and the manufacturer has altruistic preference (Model *A*), respectively. The game-theoretical models with different fairness preferences, comparative, and numerical analyses are used to put forward the impacts of consumers’ channel preference and CEA on pricing, profits, and utilities, and to identify the differences in decisional outcomes between the three models. The results indicate that CEA always contributes to developing the green market while adversely affecting common products. Moreover, consumers’ channel preferences might enable the manufacturer and retailer to enhance profitability under certain conditions. The findings also reveal that manufacturer’s fairness concerns can possibly increase the demand for green products but impair the overall performance of the supply chain in general. Moreover, while the manufacturer’s altruistic preference benefits the retailer’s profits, it has a detrimental effect on the performance of the green supply chain. The practical implications of this research come to promote green consumption and increasing consumer awareness of environmental protection are effective ways to develop a green supply chain. It is also important to note that in order to maintain the durability and stability of the sup-ply chain, the manufacturer must maintain a moderate level of fairness preference behaviors so that downstream retailers will remain enthusiastic about establishing long-term relationships.

## 1. Introduction

In light of the growing interest in environmental protection and the advent of innovative green technologies, governments and firms, as well as individual citizens, are becoming increasingly aware of environmentally friendly consumption and sustainability [1,2,3]. It has been observed that consumers are more inclined to purchase green products owing to their environmental awareness, regardless of the cost [4]. In terms of sustainability, green supply chains can be characterized as being socially, environmentally, and economically beneficial. In other words, the promotion and implementation of green ideas and sustainable development are relevant for social, economic, and environmental performance improvement [5,6].

The scope of our study covers the supply chain perspective, the business perspective, and the company perspective on green performance. First, from the perspective of the supply chain, green production and green consumption are critical components of establishing environmental protection and sustainability objectives. Second, in terms of the business side, this involves the design and use of environmentally friendly materials, and the development of waste product recycling programs to drastically reduce carbon emissions, which is also an essential step in the development of a circular economy [7]. Third, enterprises view consumer attention to the environment as a marketing opportunity. By setting a strategic goal related to environmental protection and sustainability, an enterprise can demonstrate its social responsibility, thereby establishing a reputation as a leading environmental protection enterprise, attracting more green consumers, increasing market competitiveness, and improving enterprise performance. Integrating these initiatives can significantly alleviate the extent of environmental damage and, in the long run, enhance economic benefits and social sustainability [8].

It has been advanced that, to achieve sustainable development of supply chains, it is necessary to expand investment and production in green practices [9,10,11]. Consumers’ attention to environmental sustainability is conducive to promoting green development and improving the environment [12,13,14]. Thus, numerous enterprises have initiated green product manufacturing as part of their operational strategies [15,16]. For instance, as a result of implementing green practices, the Beijing Automotive Industry Corporation (BAIC) achieves the design, production, recycling, and utilization of automotive products in compliance with environmental laws and requirements, while at the same time reaching the objectives of encouraging high-quality suppliers, improving product quality, declining product costs, reducing product resource consumption, and minimizing the environmental impact [17].

Given the importance of green activities and sustainability in supply chains, green thinking and practices are progressively becoming a significant trend for corporations to employ in supply chain management. It is common for products to be classified as green or non-green based on factors such as the use of eco-friendly materials and green technologies during the production process. Green products will become more appealing to consumers as they become more aware of global environmental sustainability [18]. Accordingly, it is imperative to investigate the pricing policies for green products and their impact on the supply chain so that businesses can accomplish their sustainable development targets [19]. Additionally, the strong growth of e-commerce and information technology has had a substantial impact on consumers’ purchasing habits and has altered the supply chain marketing approaches of numerous manufacturers [20,21]. In the modern marketplace, manufacturers have switched to multi-channel strategies, including opening direct selling channels or a combination of both online and offline retail practices [22,23]. Pakdel Mehrabani and Seifi (2021) [24] believed that consumers’ channel preferences play an essential role in manufacturers’ decisions to adopt online channels.

Although many corporations engage in sustainable development as a future development strategy, decision-makers are not always perfectly rational when it comes to implementation, and their decisions are guided not only by the pursuit of the maximization of benefits but also by their decision-making preferences [25], such as fairness concerns and altruistic preferences [26,27]. Du et al. (2017) [28] summarized that fairness concerns were conducive to the long-term development of green innovation. Further, Wang et al. (2021) [29] showed that altruistic preference improves the follower manufacturers’ profits and system efficiency in a retailer-led supply chain but reduces retailers’ profits. However, there are relatively few studies on altruistic preferences in green supply chains, and no scholars have considered the pricing policies in green dual-channel supply chains from the standpoints of fairness concerns and altruistic preferences, combined with consumers’ channel preferences and environmental awareness.

This study develops a green dual-channel supply chain model that includes one manufacturer and one retailer predicated upon the aforementioned analysis, taking CEA and consumers’ channel preferences into account when estimating the market demand. Due to the fact that the market is populated by both environmentally friendly and ordinary consumers, the manufacturer produces both green and common products simultaneously to meet the needs of the two groups. They wholesale common products to the retailer, whereas selling higher-priced green products directly to consumers. The optimal pricing strategies for both common and green products are examined in three models in which the manufacturer is fairness-neutral, fairness-concerned, or has an altruistic preference, respectively. In three situations, changes in price offerings and the influence of customers’ different preferences on product prices and profits are examined. By providing a theoretical foundation, manufacturers are able to set pricing policies that take fairness preferences into account, aiming to advance enterprises’ green development and ultimately encourage environmental sustainability. This study seeks to address three research questions: (1) How do the three models with and without fairness preferences differ in their price decision-making processes for common and green products (fairness concerns and altruistic preferences)? (2) How do the profits and utilities of the retailer and manufacturer change as a consequence of CEA and consumer channel preferences in a green dual-channel supply chain? (3) Taking into account the different types of fairness preferences of the manufacturer, what are the optimal outcomes and benefits of a green dual-channel supply chain?

To answer these questions, this study compares and analyzes the pricing decisions under different scenarios. As it stands, a great part of the previous literature only considered consumers’ singular preferences. This study scope extends to thoroughly incorporate customers’ dual preferences into market demand to construct the model more realistically, more accurately reflect the market demand, and enhance the model’s efficacy. This study seeks to examine the consequences of manufacturers’ fairness concerns and altruistic preferences, consumers’ channel preferences, and CEA on pricing decisions, profits, and utilities by applying the backward induction approach in the green supply chain. Further, this work aims to demonstrate how the different preferences of the manufacturer and consumers affect the performance and sustainability goals of green supply chains. This is accomplished by developing a basic model excluding fairness preferences, as well as two game-theoretic models with manufacturers’ differing fairness inclinations to determine optimal pricing policies and profitability. Consequently, this study is an innovative attempt to evaluate the impacts of fairness concerns, altruistic preferences, consumers’ channel preferences, and CEA on pricing strategies in sustainable and regular supply chains, respectively.

Our results indicate that CEA is always advantageous for expanding green production, regardless of the presence of different preferences of the manufacturer and consumers. However, the impacts of channel preferences and the manufacturer’s fairness preferences depend on different scenarios. Despite the fact that retailers are likely to benefit from the manufacturer’s altruistic preference, fairness preference behaviors of manufacturers are incompatible with the development of green and sustainable supply chains in the long run, eventually leading to lower benefits for the manufacturer than in a fairness-neutral situation.

The research in this study has contributed to the existing studies in several aspects. Firstly, we propose pricing policies for green dual-channel supply chains in different scenarios with the goal of enhancing and expanding green supply chain research. Secondly, regardless of whether manufacturers have divergent preferences regarding fairness, we analyze the impact of CEA and consumers’ channel preferences on pricing outcomes and profits. Thirdly, this research is capable of providing supply chain enterprises with pricing decision-making tools when a fairness-minded manufacturer maintains two channels to sell products of variable quality. Finally, by comparing the three scenarios, we demonstrate the joint interaction of different consumer preferences on pricing decisions in different scenarios. This also shows the importance of involving fairness concern and altruistic preference behaviors in pricing decisions in a green dual-channel supply chain.

The remainder of the work is organized in the following manner. Section 2 provides a brief overview of the related literature. Section 3 states the investigated problem, followed by the assumptions. Section 4 proposes the game model, identifies the optimal pricing policies, and carries on a comparative analysis. Section 5 demonstrates the numerical results and discussion. Section 6 concludes the main findings, useful implications, and future directions.

## 2. Literature Review

The literature is reviewed in four segments: pricing decisions in green supply chains, consumers preferences in supply chains, fairness preferences in supply chains, and research gaps in the discussed literature and objectives, as will be presented in the following segments. 

### 2.1. Pricing Decisions in Green Supply Chains

As public conceptions of sustainable development gradually mature, governments and enterprises currently tend to prioritize green supply chains [30,31]. The choice to adopt green supply chain practices provides a greater possibility for businesses to enhance and realize their sustainable objectives from social and environmental perspectives on the premise of without influencing economic growth. Decision-makers are increasingly paying attention to the development of environmentally friendly products and green supply chains [32,33]. For instance, Das et al. (2022) [34] designed a dual-channel supply chain considering the greenness level of products and demonstrated that the decentralized model held higher retail prices and a lower greenness level. Zhang et al. (2021) [35] captured that the pricing decisions depended on the consumers’ sensitivity to greening levels. Yang and Gong (2021) [36] considered reciprocal preferences and proposed an optimal scheme based on green production and pricing levels. Gao et al. (2020) [37] examined dual-channel green supply chain management for green products using two different green technologies. Qin et al. (2021) [38] asserted that to reduce the cost burden on manufacturers for research and development of green products, retailers should actively undertake part of the cost so that manufacturers can pay less attention to fairness concerns. This would be conducive to chain members’ benefits. Mondal and Giri (2021) [39] studied the government’s impact on decisions that optimize green performance within closed-loop supply chains and strengthen the greening levels and execution through contracts. Jamali and Rasti-Barzoki (2018) [40] examined the pricing and determination of greenness in dual-channel supply chains in which a green product was compared with a non-green product. Shi et al. (2021) [18] studied advertising and green production strategies for green and non-green products and verified that the green advertising decision was likely to be the most effective.

### 2.2. Consumers’ Preferences in Supply Chains

In light of the growing emphasis placed on environmental issues, the impact of CEA on green supply chains has attracted the attention of scholars. Generally, environmental awareness refers to the awareness and commitment to deal with environmental issues, which are impacted by numerous factors, including policy, regulation, education, and green advertising initiatives [41]. Several studies have shown that consumers have the motivation to pay additional prices for environmentally friendly products. This is because consumers increasingly aim to remain environmentally sustainable through green consumption [42,43]. Takahashi (2021) [44] revealed that delivering information to areas where consumers have high environmental awareness generated a significant positive impact on the sales of environmentally friendly products. Liu et al. (2021) [45] found that the greener consumers’ consumption patterns, the more profits they make to supply chain members. Similarly, Xu et al. (2020) [46] demonstrated through an empirical study that CEA has positive effects on consumers’ attitudes, consumption patterns, perceptions of behavioral control, and willingness to pay. Shao and Liu (2022) [47] demonstrated that green supply chains are more likely to be profitable when consumers are environmentally aware. Wang and Hou (2020) [48] identified the effect of green consumption awareness on members’ optimal decisions and the rates at which optimal price strategies can be adjusted for heterogeneous green supply chains. Qiao et al. (2021) [49] found that a higher CEA level may motivate companies to continue their environmental commitments. However, this may not always result in improved total carbon emission reductions, owing to the increased demand for the products. Heydari et al. (2020) [50] provided a theoretical view of channel coordination while incorporating CEA and proposed a hybrid greening cost and revenue-sharing contract to implement channel coordination. Zhang et al. (2019) [51] paid more attention to the impacts of CEA and retailers’ favorable and unfavorable fairness concerns. Their findings supported that CEA affected the results differently based on the retailer’s concerns about fairness.

With the growth of online channels, both the competition between offline and online channels and the consumers’ preferences have contributed to the increasing volatility of market demand [52,53]. Mehrabani and Seifi (2019) [54] explored the impact of consumers’ channel preferences on manufacturers’ adoption of online channels. They showed that, within a Pareto range, both manufacturers and retailers could benefit from the new distribution channel. Wang et al. (2022) [55] proposed a dual-channel supply chain model in which a risk-neutral manufacturer and a risk-averse retailer are combined with channel-oriented consumers. Ke and Liu (2017) [56] showed that consumers’ preferences for direct selling channels negatively affected a retailer’s profits, whereas suppliers’ profits are not. With an increase in consumers’ preference for direct selling channels, suppliers’ profits first increase but subsequently decrease. According to Zheng et al. (2021) [57], remanufacturers should consider the influence of consumers’ channel preferences, as well as the competition among channels, when deciding whether and when to offer online recycling. Moreover, Meng et al. (2021) [58] illustrated that green products are positively affected by consumers’ green preferences and online consumption preferences, with or without government subsidies.

### 2.3. Fairness Preferences in Supply Chains

Fairness preferences, including fairness concerns and altruistic preferences, have become an integral part of supply chain connections and activities [59,60,61]. Fairness concern issues have been considered in supply chain models in plenty of studies. Jian et al. (2021) [62] conducted a profound analysis of the pricing policies within supply chains, demonstrating that manufacturers’ concern for fairness harms retailers’ sales efforts and is incompatible with the development of a healthy supply chain. Wang et al. (2020) [63] verified that manufacturers’ reactions to fairness concern issues would lead to a reduction in green levels and efficiency. Toktaş-Palut (2021) [64] indicated that the greater the sensitivity of customers to greenness, the more likely manufacturers were to make more environmentally friendly products, thereby exerting favorable impacts on green demand and profitability. Consequently, manufacturers’ focus on fairness concerns increased profits for manufacturers and supply chains [65]. Moreover, Huang et al. (2022) [66] found that fairness concern behaviors of online platforms reduced the recycling rate and profits of supply chain members, which implied that fairness concern increased the profit gaps between manufacturers and platforms. Niu et al. (2017) [67] advanced that retailers’ advantageous and disadvantageous fairness concerns reduced manufacturers’ incentives to launch online channels.

Some scholars have also integrated altruistic preferences into supply chains. Wan et al. (2020) [68] investigated the impact of the altruistic preferences of managers and consumers’ preferences for low-carbon consumption on decisional behaviors at all levels. They discovered that decision-makers’ altruistic preferences had varying consequences on the supply chain under business and agency models. Huang et al. (2019) [69] pointed out that the altruistic preferences of decision-makers are advantageous to the supply chain and green products. Rong and Xu (2022) [70] derived and evaluated the optimal solutions for stakeholders in order to assess the influence of altruism and government subsidies on a global green supply chain operating under a dynamic tariff regime. Liu et al. (2021) [71] demonstrated that the moderate altruistic preference behavior of e-platforms is advantageous for developing sustainable supply chains. Ma et al. (2021) [72] integrated reciprocal, altruistic, and consumers’ channel preferences into an online to offline product-service supply chain decision. They realized that consumers’ online channel preference is advantageous to total supply chain efficiency, and different levels of altruism preference have different effects on supply chain decisions. They concluded that additional welfare was only created when the manufacturer and retailer had pure altruism preferences.

### 2.4. Research Gap

Table 1 summarizes and compares the main contributions of the related studies.

According to the reviewed literature, most green supply chain management studies concentrate on pricing outcomes, the performance of chain members, and consumer preferences. It is noteworthy that supply chain participants always have a bounded rational attitude towards dealing with actual problems; that is, they need to emphasize their own interests while making decisions and attach importance to whether other participants are fairly distributed. Although several studies have examined the effects of fairness concerns and altruistic preferences, they have primarily examined non-green or single-channel supply chains. Few results regarding the relationship between fairness concern, altruistic preference, consumers’ channel preference, and CEA in a green dual-channel supply chain. Moreover, few studies have investigated the coexistence of common and green products in dual-channel supply chains, focusing instead on marketing a single product. Likewise, the existing literature frequently assesses the influence of a single behavior or preference factor of consumers rather than comprehensively considering the consumers’ multiple influences. However, many companies sell both common and green products through dual channels, and consumers’ behaviors and preferences are generally more diverse. A combination of factors can influence the performance and efficiency of a green supply chain. The research on this topic remains insufficient. We have therefore decided to conduct this study to fill the gaps by integrating different fairness preferences into a green dual-channel supply chain model and analyzing their impact on optimal results and profits. Our findings assist enterprises in determining optimal outcomes and green practices with different fairness preferences and consumer preferences and also provides managerial implications.

## 3. Problem Description and Assumptions

This paper targets a green dual-channel supply chain involving a manufacturer and a retailer. By incorporating consumers’ channel preferences and CEA, this study determines the pricing policies of common products and green products under the consideration of the different fairness behaviors of the manufacturer. The manufacturer is in charge of green Research and Development (R&D), manufactures two types of products, and puts them on the market separately through two channels. Specifically, the manufacturer wholesales common products to the retailer through the offline retail channel at price $\omega_{n}\;$ and provides green products to consumers through its online direct sales channel at price $p_{g}$. The retailer sells common products to consumers through a brick-and-mortar channel at price $p_{n}$, then $p_{n}>\omega_{n}$. $c_{g}$ and $c_{n}$ are the costs for green products and common products, respectively. Since there are additional costs involved in manufacturing green products, such as green R&D costs and high-priced environmentally friendly materials costs, this study considers that the cost of green products is greater than that of common products, namely $c_{g}>c_{n}$. Moreover, asymmetrical and unfair green cost inputs and corresponding returns of manufacturers and retailers may be encountered during the practice of green supply chain management, thereby triggering the fairness concern mechanism, which may lead to a change in the final decision variables as a consequence [63,73]. Basically, manufacturers are more attentive to the profitability of their operations and the fairness of channel distribution because they invest a substantial amount of resources in R&D as well as in the production of common and green products [65]. Therefore, we believe that manufacturers are more inclined toward fairness preference behaviors. This study respects the following order: the manufacturer determines the wholesale price first, then the retailer determines the retail price in accordance with the wholesale price. This study employs a variety of notations, which are detailed in Table 2.

Prior to setting up the suggested model, a few assumptions are put forward: some are intended to enhance the model’s realism, and others are used for simplification purposes. All of them are nonetheless inspired by previous studies in this field [18,35,58]. Hence, the following assumptions are made in view of creating a sound mathematical model.

**Assumption** **1.** *To simplify the model, this paper does not examine horizontal competition, and it simply describes the case with a single manufacturer and a single retailer. The manufacturer has a predominant rule with the decision priority, and each individual is typically permitted to purchase a single unit of product*.

**Assumption** **2.** *The costs associated with green R&D are influenced by the greenness of a product and increase exponentially with it. In line with previous work [19,74], the cost of green R&D is assumed to be* $\frac{1}{2}\beta \tau^{2}$ *, where* β *represents the cost coefficient for green R&D,* β>0 *, and* τ *represents the greenness level of the green product.*

**Assumption** **3.** *For the sake of simplicity, it is believed that all information is symmetrical to the channel members, regardless of information asymmetry.*

**Assumption** **4.** *As a result of consumption preferences for green products, consumers with a greater environmental awareness are more likely to buy green products. Since the consumer’s offline channel preference coefficient is assumed to be*δ (0≤δ≤1)*, then we have the online channel preference coefficient*1−δ*. As the market demand in this paper is affected by some relevant factors such as: consumer preferences, the greenness of products and their prices, referring to the previous literature [51,63], it can be expressed as a linear equation:*

(1) $$\;d_{n}=\delta a-kp_{n}+\gamma p_{g}+\left(1-\eta \right)\tau$$

(2) $$\;d_{g}=\left(1-\delta \right)a-kp_{g}+\gamma p_{n}+\eta \tau$$

*where we assume that*k*and*γ*satisfy*k>γ>0*, implying that a product’s demand is more sensitive to changes in the price of that product than the price of its competitors. Parameter*k*is normalized to one in the following for brevity’s sake, thus, we have*0<γ<1*. It is evident that product demand decreases as its price increases, whereas an increase in demand occurs as its competitor’s price increases. Meanwhile, η refers to consumer environmental awareness (CEA) and*0≤η≤1*. CEA reflects consumers’ willingness to purchase green products. Higher CEA indicates that consumers are more likely to purchase green products. There are two main reasons for the adoption of linear demand functions. In the first place, the demand curve can be well described by a linear structure [75,76]. The second reason for the widespread use of linear models in operational management literature is that they are tractable and highly parsimonious [51,77]*.

**Assumption** **5.** *It is widely accepted by the consumers that the two types of products (common products and green products) are substitutes for one another, thus they are competitors. For simplicity, we consider that these two products are the only products available to the consumer on the market.*

**Assumption** **6.** *The manufacturer has sufficient capacity to meet all demand for green and common products, and there are no lost sales or backorders.*

The Stackelberg game is also known as the “leader-follower model”. The Stackelberg game model is primarily composed of leaders and followers. The objective of the game is to maximize revenue while considering the strategies of both leaders and followers. The Stackelberg competition model is a price leadership model in which firms differ in the order of actions. The leader has greater decision power; therefore, the output decision follows this pattern: the leader firm decides an output, then the follower firm observes this output, and then determines its own output based on the output of the leader firm. As noted, the leading manufacturer will consider the response of the follower when deciding its output—this means that the leader can understand the reaction function of the follower and then incorporate this reaction into its decisions. In addition, since we assume that manufacturers are not perfectly rational decision-makers, they may have different preferences regarding fairness behavior. To make the scope of this study more comprehensive, our model is expanded to include the cases of manufacturer fairness neutrality, fairness concern, and altruistic preference. Through comparative and numerical analyses, we are able to obtain the relationship between prices, demand, and profits under different conditions.

## 4. Model Construction and Analysis

This section examines the following situations: Models *N*, *F*, and *A.* The Model *N* is taken as a benchmark model and extended to analyze the effects of fairness concern and altruistic preference behaviors on product prices, profits, and utilities. In accordance with the assumptions outlined above, the profit functions of the manufacturer and retailer can be described as follows:

(3) $$\pi_{r}=\left(p_{n}-\omega_{n}\right)d_{n}$$

(4) $$\pi_{m}=\left(\omega_{n}-c_{n}\right)d_{n}+\left(p_{g}-c_{g}\right)d_{g}-\frac{1}{2}\beta \tau^{2}$$

### 4.1. A Benchmark Model (N)

For the purpose of examining the effects of fairness concerns on initial pricing and decisions, we first propose a scenario in which the manufacturer and the retailer are perfectly rational. During the decision process, the manufacturer and retailer decide their prices in order to attain profit maximization. The following propositions describe the optimal results for Model *N*.

**Proposition** **1.** *In Model N, from Equations (1)–(4), the optimal equilibrium solutions are as follows:*

$$\omega_{n}^{N*}=\frac{Y_{1}}{2\left(1-\gamma^{2}\right)}+\frac{c_{n}}{2},\;p_{g}^{N*}=\frac{Y_{2}}{2\left(1-\gamma^{2}\right)}+\frac{c_{g}}{2},\;p_{n}^{N*}=\frac{a\left(\delta \left(\gamma -1\right)\left(3+\gamma \right)-2\gamma \right)+Y_{3}}{4\left(1-\gamma^{2}\right)}+\frac{c_{g}\gamma +c_{n}}{4}$$

*The optimal solutions can be substituted into Equations (1)–(4) to obtain the demand and profit values under the optimal choices of prices, as follows:*

$$d_{n}^{N*}=\frac{T_{0}}{4},\;d_{g}^{N*}=\frac{a\left(2+\delta \left(\gamma -2\right)\right)+c_{n}\gamma +c_{g}\left(\gamma^{2}-2\right)+2\eta \tau +\gamma \tau -\eta \gamma \tau }{4},\;\pi_{r}^{N*}=\frac{T_{0}^{2}}{16}$$

$$\;\;\;\;\;\;\;\;\;\pi_{m}^{N*}=\frac{2a\tau T_{1}-a^{2}T_{2}-\tau^{2}T_{3}}{8\left(1-\gamma^{2}\right)}+\frac{c_{n}^{2}-2c_{n}\left(\delta a+c_{g}\gamma +\tau -\eta \tau \right)-c_{g}T_{4}}{8}$$

*where,*$\;Y_{1}=a\left(\delta +\gamma -\delta \gamma \right)+\left(1-\eta +\eta \gamma \right)\tau$, $Y_{2}=a\left(1-\delta +\delta \gamma \right)+\left(\eta +\gamma -\eta \gamma \right)\tau$, $Y_{3}=\left(\gamma^{2}-3-\eta \left(\gamma -1\right)\left(3+\gamma \right)\right)\tau$, $T_{0}=\delta a-c_{n}+c_{g}\gamma +\tau -\eta \tau$, $T_{1}=\delta \left(1+\eta \left(\gamma -3\right)-\gamma \right)\left(\gamma -1\right)+2\eta \gamma -2\left(\eta +\gamma \right)$, $T_{2}=2+\delta \left(4+\delta \left(\gamma -3\right)\right)\left(\gamma -1\right)$, $T_{3}=1+\eta^{2}\left(\gamma -3\right)\left(\gamma -1\right)-2\eta {\left(\gamma -1\right)}^{2}+\gamma^{2}+4\beta \left(\gamma^{2}-1\right)$, $T_{4}=2a\left(2+\delta \left(\gamma -2\right)\right)+c_{g}\left(\gamma^{2}-2\right)+4\eta \tau -2\left(\eta -1\right)\gamma \tau$.

For Proofs of all propositions, see Appendix A.

**Proposition** **2.** *The number of environmentally conscious consumers is indeed increasing year by year, but generally speaking, the number of consumers willing to buy green products is always lower than that for common products due to the consideration of price. Therefore, to make the model more realistic and feasible and to improve the efficiency of the decisions, we assume that the demands*$d_{n}$*and* $d_{g}\;$*need to satisfy the constraint condition of*$0\leq d_{g}\leq d_{n}$*and the following is the proposed relationship between competition in the market:*

 *(1)*  *When*0≤η≤min{1,M}*, the demand for green products is zero, where*$M=\frac{2a-2c_{g}-2a\delta +c_{n}\gamma +\delta a\gamma +c_{g}\gamma^{2}+\gamma \tau }{\left(\gamma -2\right)\tau }$.
 *(2)*  *When*max{0,M}≤η≤min{1,N}, *both green and common products are in demand, where*$N=\frac{2a+c_{n}-2c_{g}-3\delta a+c_{n}\gamma -c_{g}\gamma +\delta a\gamma +c_{g}\gamma^{2}-\tau +\gamma \tau }{\left(\gamma -3\right)\tau }$

From Proposition 2, when 0≤η≤min{1,M}, the demand for green products is zero. The reason for this is that when the CEA is significantly low, the willingness of consumers to purchase green products is extremely low as well, so the market for green goods will be completely ignored. When max{0,M}≤η≤min{1,N}, both common products and green products are in demand; this is due to the improvement in public awareness of environmental protection, the purchase intention of some consumers for green products equally increases, such that both common products and green products have corresponding markets.

**Corollary** **1.** *The impacts of CEA on prices, demand, and profits in Model N are shown as:* $\frac{\partial \omega_{n}^{N*}}{\partial \eta }<0$, $\frac{\partial p_{g}^{N*}}{\partial \eta }>0$, $\frac{\partial p_{n}^{N*}}{\partial \eta }<0$, $\frac{\partial d_{n}^{N*}}{\partial \eta }<0$, $\frac{\partial d_{g}^{N*}}{\partial \eta }>0$, $\frac{\partial \pi_{r}^{N*}}{\partial \eta }<0$, $\frac{\partial \pi_{m}^{N*}}{\partial \eta }>0$.

**Corollary** **2.** *The influences of consumers’ channel preference on decisions under Model N are as follows:* $\frac{\partial \omega_{n}^{N*}}{\partial \delta }>0$, $\frac{\partial p_{g}^{N*}}{\partial \delta }<0$, $\frac{\partial p_{n}^{N*}}{\partial \delta }>0$, $\frac{\partial d_{n}^{N*}}{\partial \delta }>0$, $\frac{\partial d_{g}^{N*}}{\partial \delta }<0$, $\frac{\partial \pi_{r}^{N*}}{\partial \delta }>0$, $\frac{\partial \pi_{m}^{N*}}{\partial \delta }<0$.

For Proofs of all corollaries, see Appendix A.

Corollary 1 and Corollary 2 analyze how CEA and consumers’ channel preference affect the wholesale price, retail prices, demand, and profits of supply chain members under Model *N*. Corollary 1 shows that, as CEA increases, the optimal wholesale price, retail price, and demand for common products decrease, whereas the direct sale price of green products and demand increase. A larger *η* implies that consumers have a stronger environmental awareness, leading to a higher greenness level for green products. This results in a reduction in profits for retailers who only sell common products, whereas manufacturers benefit from having access to both common and green products.

It follows from Corollary 2 that as consumers’ preference for offline channels increases, the optimal wholesale price, retail price, and demand for common products also increase. Due to the fact that green products are only available online, the channel preference of consumers (*δ*) is negatively affecting the sales of green products. As a result, as consumers prefer offline channels, the demand for common products expands, and the retailer raises the retail price to maximize profits. Consequently, the demand for green products decreases, and the manufacturer typically boosts sales by reducing the direct sale price of green products, resulting in a lower yield.

### 4.2. Decisions with Fairness Concern (F)

In this subsection, we study pricing policy in a green supply chain characterized by a fairness-concerned manufacturer and a fairness-neutral retailer. In addition to considering their own interests, manufacturers also pay great attention to the fairness of profit distribution in the supply chain. It is likely that if the retailer’s profit exceeds or falls below the manufacturer’s profit, the manufacturer will perceive this as unfair and may change its product pricing and production quantity or even reduce green production investments to improve the fairness concern utility. The manufacturer employs retailer profits as a reference point, and its fairness concern utility decreases when the profit of the retailer becomes higher, and vice versa. According to the study by Jian et al. (2021) [62], without reducing generality, the utility of the manufacturer can be defined as below:

(5) $$u_{m}^{F}=\pi_{m}^{F}-\lambda \left(\pi_{r}^{F}-\pi_{m}^{F}\right)$$

where λ is the fairness concern level of the manufacturer towards the retailer, and λ∈(0,1). Substituting Equations (1)–(4) into Equation (5), the manufacturer’s utility function can be identified as:

(6) $$\begin{array}{c} u_{m}^{F}=\left(1+\lambda \right)(\left(\omega_{n}^{F}-c_{n}\right)\left(\delta a-p_{n}^{F}+\gamma p_{g}^{F}+\left(1-\eta \right)\tau \right)+\left(p_{g}^{F}-c_{g}\right)\left(\left(1-\delta \right)a-p_{g}^{F}+\gamma p_{n}^{F}+\eta \tau \right)\\ -\frac{1}{2}\beta \tau^{2})-\lambda \left(p_{n}^{F}-\omega_{n}^{F}\right)\left(\delta a-p_{n}^{F}+\gamma p_{n}^{F}+\left(1-\eta \right)\tau \right) \end{array}$$

As in Proposition 1, backward induction yields optimal results for Model *F*, which are provided in Proposition 3.

**Proposition** **3.** *In Model F, based on the above profits and utility functions, the optimal solutions are shown as follows:*

$$\omega_{n}^{F*}=\frac{a\lambda \left(\delta \left(\gamma +4\right)\left(\gamma -1\right)-3\gamma \right)-2Y_{1}+\lambda Z_{1}}{2\left(\gamma^{2}-1\right)\left(2+3\lambda \right)}+\frac{c_{g}\gamma \lambda +2c_{n}\left(1+\lambda \right)}{2\left(2+3\lambda \right)},\;p_{g}^{F*}=\frac{Y_{2}}{2\left(1-\gamma^{2}\right)}+\frac{c_{g}}{2}$$

$$p_{n}^{F*}=\frac{Y_{3}+\left(2\gamma^{2}-5-\eta \left(\gamma -1\right)\left(5+2\gamma \right)\right)\tau \lambda -a\gamma \left(2+3\lambda \right)+aZ_{2}}{2\left(\gamma^{2}-1\right)\left(2+3\lambda \right)}+\frac{c_{g}\gamma \left(1+2\lambda \right)+c_{n}\left(1+\lambda \right)}{2\left(2+3\lambda \right)}\;$$

*Then, the demand and profit values under the optimal choices of prices can be further obtained as:*

$$d_{n}^{F*}=\frac{\left(1+\lambda \right)T_{0}}{2\left(2+3\lambda \right)},\;d_{g}^{F*}=\frac{\gamma \left(1+\lambda \right)c_{n}+\gamma \left(1+2\lambda \right)\left(\tau -\eta \tau +\gamma c_{g}\right)+aZ_{3}}{2\left(2+3\lambda \right)}+\frac{a+\eta \tau -c_{g}}{2},\;\pi_{r}^{F*}=\frac{{\left(1+\lambda \right)}^{2}T_{0}^{2}}{2{\left(2+3\lambda \right)}^{2}}$$

$$\pi_{m}^{F*}=\frac{\left(c_{g}\gamma \lambda +2c_{n}\left(1+2\lambda \right)\right)\left(1+\lambda \right)T_{0}}{\;4{\left(2+3\lambda \right)}^{2}}+\frac{\left(1+\lambda \right)T_{0}\left(2Y_{1}+a\lambda \left(3\gamma -\delta \left(\gamma +4\right)\left(\gamma -1\right)\right)-\tau \lambda Z_{1}\right)}{4\left(\gamma^{2}-1\right){\left(2+3\lambda \right)}^{2}}-\frac{\beta \tau^{2}}{2}-\;\frac{Y_{2}\left(\left(a+\eta \tau -c_{g}\right)\left(2+3\lambda \right)+\gamma \left(1+\lambda \right)c_{n}+\gamma \left(1+2\lambda \right)\left(\tau -\eta \tau +\gamma c_{g}\right)+aZ_{3}\right)}{4\left(\gamma^{2}-1\right)\left(2+3\lambda \right)}\;,$$

$$\begin{array}{ll} u_{m}^{F*}=({\left(1+\lambda \right)}^{2}c_{n} & (c_{n}+2\tau \eta -2\tau ))/(-4(2+3\lambda ))+(1+\lambda )(T_{3}\tau^{2}+\lambda (1-6\beta -2\eta +4\eta^{2}+6(1-\eta )\eta \gamma +2(3\beta^{2}\\ & +{\left(\eta -1\right)}^{2}\gamma^{2})\tau^{2})+a^{2}(2+3\lambda +\delta (\gamma -1)(4+6\lambda +\delta Z_{4}))+2a(2+3\lambda +Z_{3})+\tau ((\eta +\gamma -\eta \gamma )(2\\ & +3\lambda )-\delta (\gamma -1)(1+\lambda -\gamma (1+2\lambda )+\eta Z_{4})))/(4(1-\gamma^{2})(2+3\lambda ))+(1+\lambda )(c_{g}^{2}(-2-3\lambda +\gamma^{2}(1\\ & +2\lambda ))+2c_{g}(\eta \tau (2+3\lambda )+c_{n}\gamma (1+\lambda )+(1-\eta )\gamma \tau (1+2\lambda ))+2ac_{n}\delta (1+\lambda )+2ac_{g}(2+3\lambda \\ & +Z_{3}))/(-4(2+3\lambda )) \end{array}$$

*where,*$Z_{1}=\tau \left(\gamma^{2}-4-\eta \left(\gamma -1\right)\left(\gamma +4\right)\right)$*,*$\;Z_{2}=\delta \left(\gamma -1\right)\left(3+\gamma +5\lambda +2\gamma \lambda \right)$*,*$\;Z_{3}=\delta \left(\gamma -2-3\lambda +2\gamma \lambda \right)$*,*$\;Z_{4}=\gamma -3+2\left(\gamma -2\right)\lambda$.

**Corollary** **3.** $\omega_{n}^{F*}$, $p_{n}^{F*}$, $d_{n}^{F*}$*, and* $\pi_{r}^{F*}$*are negatively related with η, whereas* $p_{g}^{F*}$*and* $d_{g}^{F*}$*are positively related with η, which can be shown as* $\frac{\partial \omega_{n}^{F*}}{\partial \eta }<0$, $\frac{\partial p_{n}^{F*}}{\partial \eta }<0$, $\frac{\partial d_{n}^{F*}}{\partial \eta }<0$, $\frac{\partial \pi_{r}^{F*}}{\partial \eta }<0$, $\frac{\partial p_{g}^{F*}}{\partial \eta }>0$, and $\frac{\partial d_{g}^{F*}}{\partial \eta }>0$.

**Corollary** **4.** *Based on Proposition 3, the following relationships hold:*$\frac{\partial \omega_{n}^{F*}}{\partial \delta }>0$, $\frac{\partial p_{n}^{F*}}{\partial \delta }>0$, $\frac{\partial d_{n}^{F*}}{\partial \delta }>0$, $\frac{\partial p_{g}^{F*}}{\partial \delta }<0$, $\frac{\partial d_{g}^{F*}}{\partial \delta }<0$, $\frac{\partial \pi_{r}^{F*}}{\partial \delta }>0$.

Corollary 3 and Corollary 4 present the effects of CEA and consumers’ channel preference on optimal solutions under Model *F*. As can be seen, CEA adversely affects the optimal wholesale price, retail price, and demand for common products, as well as the retailer’s profit. It is evident that the price and demand for green products increase as *η* grows. This is because if consumers are susceptible to environmental awareness, they tend to pay a premium for green products to maintain ecological health, which means they prefer to purchase green products from online channels. This results in a decline in retail sales of common commodities, leading to a decrease in retail prices, whereas the manufacturer becomes more profitable as demand for green products increases.

In addition, consumers’ preference for the offline channel has the opposite effect on the corresponding variables as CEA. Specifically, the optimal wholesale price, retail price, and demand for common products are positively correlated with *δ*, and as a result, the profit of the retailer improves. This is similar to the outcome of Corollary 2. However, in terms of the manufacturer, the effects of *η* and *δ* on the profits and utilities are too complex to be judged due to the influence of fairness concerns. This further shows that although the fairness concern of manufacturers has no apparent influence on the changing trends of variables such as prices and demand of common and green products, it significantly influences their own profits. Therefore, we analyze the changes in the manufacturer’s profit and utility under the influence of the fairness concern coefficient in numerical analysis.

**Corollary** **5.** *The impacts of the fairness concern coefficient on wholesale price, sale prices, demand, and profits can be obtained as*:$\;\partial \omega_{n}^{F*}/\partial \lambda >0$, $\partial p_{n}^{F*}/\partial \lambda >0$, $\;\partial d_{n}^{F*}/\partial \lambda <0$, $\;\partial d_{g}^{F*}/\partial \lambda >0$, $\;\partial \pi_{r}^{F*}/\partial \lambda <0$, $\;\partial \pi_{m}^{F*}/\partial \lambda <0$.

Corollary 5 determines that when a manufacturer is fairness concerned, the wholesale and retail prices of common products are increasing with the fairness concern coefficient, resulting in a decrease in common demand and, ultimately, a decrease in retailer profits. In addition, the direct sales prices of green products remain unaffected by the fairness concern behavior. Thus, an increase in the prices of common products will encourage some consumers to switch to green products, thereby increasing the demand for green products. However, the manufacturer’s profits continue to decrease as *λ* increases. This signifies that, with a fairness concern behavior by the manufacturer, the loss of demand caused by higher pricing for common products is far greater than the growth in green products, which ultimately results in lower profits.

### 4.3. Decisions with Altruistic Preference (A)

Numerous studies have shown that, in order to maintain stability and coordination and promote sustainable development in supply chain operations, the leading members often reflect the attribute of altruistic preference to a certain extent to promote cooperation between the two sides [71,78,79]. There is a tendency among manufacturers to pay attention not only to the interests of their own companies but also to the benefits of retailers. Manufacturers contribute a portion of their profits to retailers, increase their enthusiasm to cooperate, and create a fair and just internal competitive environment through channels. In this case, the manufacturer holds wholesale pricing power and occupies a more advantageous position in the market. In order to reflect the social value of the enterprise and improve competitiveness, the manufacturer will moderate altruistic preferences as part of corporate decision making. Since the manufacturer is acting as a leader, in the absence of evidence to the contrary, the manufacturer is presumed to have altruistic intentions, whereas the retailer is altruistic neutral. Referring to the work of Huang et al. (2019) [69], in the interests of generality, the altruistic utility of the manufacturer can be defined as below:

(7) $$u_{m}^{A}=\pi_{m}^{A}+\mu \pi_{r}^{A}$$

where *μ* is the altruistic preference level of the manufacturer towards the retailer, and *μ* ϵ (0, 1). We can see that the larger the coefficient of altruistic preference, the higher the utility of the manufacturer. As in Section 4.2, the optimal solutions for Model *A* are provided in Proposition 4.

**Proposition** **4.** *In Model A, based on the aforementioned profits and utility functions, the optimal equilibrium solutions are derived as follows:*

$$\omega_{n}^{A*}=\frac{2c_{n}-\mu \gamma c_{g}}{2-\mu }+\frac{2Y_{1}+a\mu \left(\delta H_{1}-\gamma \right)+\mu \tau \left(\gamma^{2}-2-\eta H_{1}\right)}{2\left(1-\gamma^{2}\right)\left(2-\mu \right)},\;p_{g}^{A*}=\frac{Y_{2}}{2\left(1-\gamma^{2}\right)}+\frac{c_{g}}{2}$$

$$p_{n}^{A*}=\frac{1}{2}\left(c_{g}\gamma +\frac{a\left(\delta H_{1}-\gamma \right)+\tau \left(\gamma^{2}-2-\eta H_{1}\right)}{\gamma^{2}-1}+\frac{T_{0}}{\mu -2}\right)$$

*Then, the demand and profit values under the optimal choices of prices can be further expressed as:*

$$d_{n}^{A*}=\frac{T_{0}}{2\left(2-\mu \right)},\;d_{g}^{A*}=\frac{Y_{2}+c_{g}\left(\gamma^{2}-1\right)}{2}-\frac{\gamma T_{0}}{2\left(2-\mu \right)},\;\pi_{r}^{A*}=\frac{T_{0}^{2}}{4{\left(\mu -2\right)}^{2}}$$

$$\pi_{m}^{A*}=\frac{c_{n}T_{0}\left(\mu -1\right)}{2{\left(\mu -2\right)}^{2}}+\frac{T_{0}\left(2Y_{1}+\mu \left(c_{g}\gamma \left(\gamma^{2}-1\right)+a\left(\delta H_{1}-\gamma \right)+\tau \left(\gamma^{2}-2-\eta H_{1}\right)\right)\right)}{4\left(\gamma^{2}-1\right){\left(\mu -2\right)}^{2}}+\frac{\left(Y_{2}+c_{g}\left(\gamma^{2}-1\right)\right)\left(c_{g}H_{2}+aH_{4}-H_{3}\right)}{4\left(\gamma^{2}-1\right)\left(\mu -2\right)}\;$$

$$\begin{array}{ll} u_{m}^{A*}=(c_{n}^{2}\left(1-\gamma^{2}\right) & -2c_{n}\left(1-\eta \right)\left(1-\gamma^{2}\right)\tau +T_{3}\tau^{2}+c_{g}^{2}\left(1-\gamma^{2}\right)H_{2}-(\left(\eta +\gamma -\eta \gamma {)}^{2}+2\beta \left(\gamma^{2}-1\right)\right)\tau^{2}\mu +2c_{g}\left(\gamma^{2}-1\right)H_{3}\\ & +a^{2}\left(2-\mu +\delta \left(\gamma -1\right)\left(4-2\mu -\delta H_{5}\right)\right)+2a(c_{n}\delta \left(\gamma^{2}-1\right)-c_{g}\left(\gamma^{2}-1\right)H_{4}+\tau (\left(\eta \gamma -\eta -\gamma \right)\left(\mu -2\right)\\ & +\delta \left(\gamma -1\right)\left(\gamma -1-\gamma \mu +\eta H_{5}\right)))/\left(4\left(1-\gamma^{2}\right)\left(2-\mu \right)\right) \end{array}$$

*where,*$\;H_{1}=\gamma^{2}+\gamma -2$*,*$H_{2}=2+\gamma^{2}\left(\mu -1\right)-\mu$*,*$H_{3}=c_{n}\gamma +2\eta \tau +\tau \left(\left(1-\eta \right)\gamma \left(1-\mu \right)-\eta \mu \right)$*,*$H_{4}=\mu -2+\delta \left(2-\gamma +\left(\gamma -1\right)\mu \right)$*,*$H_{5}=3-\gamma +\gamma \mu -\mu$.

**Corollary** **6.** *According to Proposition 4, the following properties hold:*

 *(1)*  *if*0<μ<2/(2+γ), $\frac{\partial \omega_{n}^{A*}}{\partial \eta }<0$, *otherwise* $\frac{\partial \omega_{n}^{A*}}{\partial \eta }>0$;
 *(2)*  $\frac{\partial p_{g}^{A*}}{\partial \eta }>0$, $\frac{\partial p_{n}^{A*}}{\partial \eta }<0$, $\frac{\partial d_{n}^{A*}}{\partial \eta }<0$, $\frac{\partial d_{g}^{A*}}{\partial \eta }>0$, $\frac{\partial \pi_{r}^{A*}}{\partial \eta }<0$.

**Corollary** **7.** *The influences of consumers’ channel preferences on decisions under Model A satisfy the following relationship:*

 *(1)*  *if*0<μ<2/(2+γ), $\frac{\partial \omega_{n}^{A*}}{\partial \delta }>0$, *otherwise*$\frac{\partial \omega_{n}^{A*}}{\partial \delta }<0$;
 *(2)*  $\frac{\partial p_{g}^{A*}}{\partial \delta }<0$, $\frac{\partial p_{n}^{A*}}{\partial \delta }>0$, $\frac{\partial d_{n}^{A*}}{\partial \delta }>0$, $\frac{\partial d_{g}^{A*}}{\partial \delta }<0$, $\frac{\partial \pi_{r}^{A*}}{\partial \delta }>0$.

Corollary 6 and Corollary 7 point out the variation of decision variables under the influence of the manufacturer’s altruistic preference. Corollary 6(1) indicates that the changing of the wholesale price depends on the range of *μ*. When the altruistic preference coefficient is within a specific condition, which can be described as 0<μ<2/(2+γ), the wholesale price of common products is adversely affected by CEA. Otherwise, the wholesale price would rise with the CEA. This means that wholesale prices are clearly affected by manufacturers’ altruistic preferences, in contrast to fairness concerns. When the manufacturer’s altruistic preference exceeds a reasonable range, the wholesale price of the common product increases with an increase in *η*, which is not necessarily beneficial to the retailer. Based on Corollary 6(2), the retail price and demand for common products, along with the retailer’s profits, decline as *η* grows. Nevertheless, green products continue to benefit from the growing green market.

Corollary 7 shows that the consumers’ channel preference has different effects on the decision outcomes. Similarly, the wholesale price with altruistic preferences is determined by the range of *μ*. In other words, when 0<μ<2/(2+γ), the wholesale price increases as *δ* increases. However, if *μ* is outside this range, the wholesale price shows the opposite trend. In light of consumers’ preferences for brick-and-mortar stores, prices and sales of common products will rise, leading to a decline in the green product market. Generally, a manufacturer’s altruistic preference should be kept within a reasonable range; otherwise, an excessive transfer of benefits from the manufacturer to the retailer could threaten their own profitability and eventually lead to supply chain instability.

**Corollary** **8.** *The impacts of the altruistic preference coefficient on wholesale price, sale prices, demand, and profits can be obtained as:*$\;\partial \omega_{n}^{A*}/\partial \mu <0$, $\partial p_{n}^{A*}/\partial \mu <0$, $\partial d_{n}^{A*}/\partial \mu >0$, $\;\partial d_{g}^{A*}/\partial \mu <0$, $\partial \pi_{r}^{A*}/\partial \mu >0$, $\;\partial \pi_{m}^{A*}/\partial \mu <0$, $\partial u_{m}^{A*}/\partial \mu >0$.

Corollary 8 investigates the implications of the manufacturer’s altruistic preference for different variables. In comparison to the fairness concern coefficient, altruistic preference has the opposite effect on decision-making results. It is highlighted that the greater the manufacturer’s altruistic preference, the more beneficial it is to common product sales and the more detrimental it is to green product sales. Hence, it is possible to infer that the increase in the manufacturer’s altruistic preference benefits the retailer. However, as more consumers opt for lower-priced common products, the demand for green products declines dramatically, ultimately resulting in a reduction in the manufacturer’s profit but an increase in the manufacturers’ utility as *μ* increases.

**Corollary** **9.** *By a comparative analysis of the optimal outcomes of the three models, the relationships among optimal prices, demand, and profits can be illustrated as follows:*$\;\omega_{n}^{F*}>\omega_{n}^{N*}>\omega_{n}^{A*}$, $\;p_{n}^{F*}>p_{n}^{N*}>p_{n}^{A*}$, $\;p_{g}^{F*}=p_{g}^{N*}=p_{g}^{A*}$, $d_{n}^{F*}<d_{n}^{N*}<d_{n}^{A*}$, $d_{g}^{F*}>d_{g}^{N*}>d_{g}^{A*}$, $\pi_{r}^{F*}<\pi_{r}^{N*}<\pi_{r}^{A*}$, $\pi_{m}^{F*}<\pi_{m}^{N*}$, $\pi_{m}^{A*}<\pi_{m}^{N*}$.

In accordance with Corollary 9, the wholesale prices and retail prices of common products sold in the offline channel are highest when the manufacturer is fairness-concerned and lowest when the manufacturer is altruistic, whereas the prices of green products remain unchanged. The increase in the prices of common products leads to a decrease in demand; that is, when the manufacturer is fairness-concerned, the demand for common goods becomes the lowest, which leads to a lower profit for the retailer. When the manufacturer has an altruistic preference, the demand for common products and the retailer’s profit will increase. However, for the change in the demand for green products, the outcome is rather the opposite. Additionally, the profits of the manufacturer are lower when fairness concerns and altruistic preferences are present, indicating that manufacturers’ fairness preferences diminish their profits.

## 5. Numerical Analysis

The theoretical analysis above indicates that CEA and channel preference, as well as the fairness concern and altruistic preference behaviors, all have a significant impact on supply chain decision making and profit optimization in the three models. Several numerical examples are presented to demonstrate the consequences of these variables on optimal supply chain outcomes. Following are the assumed values for the parameters used in numerical examples: *a* = 50, *c_n_* = 10, *c_g_* = 40, *γ* = 0.5, *β* = 0.6, and *τ* = 26. The parameters of the proposed model are set to values that support the assumptions of the model.

### 5.1. Impact of CEA η

**Figure 1:**
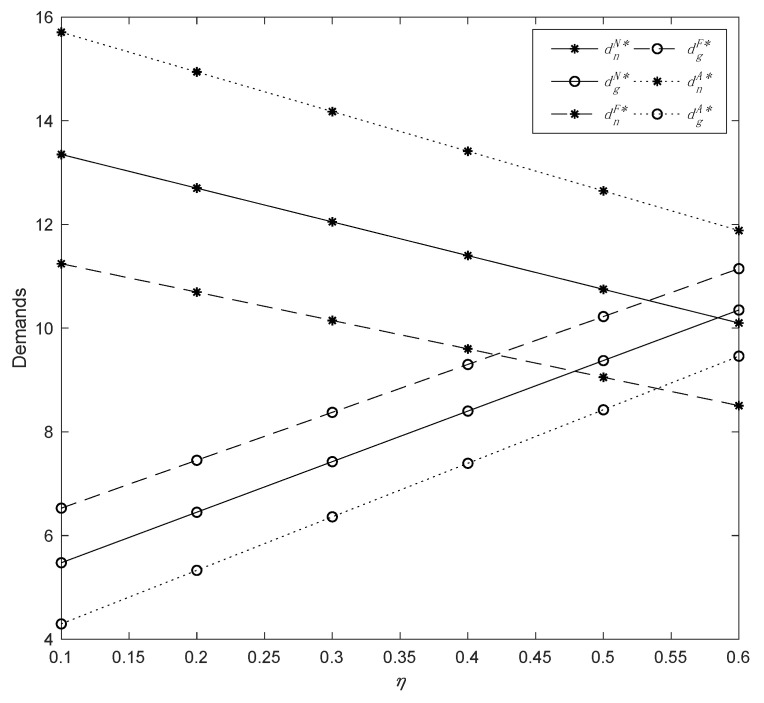
The change in demands with parameter *η*.

**Figure 2:**
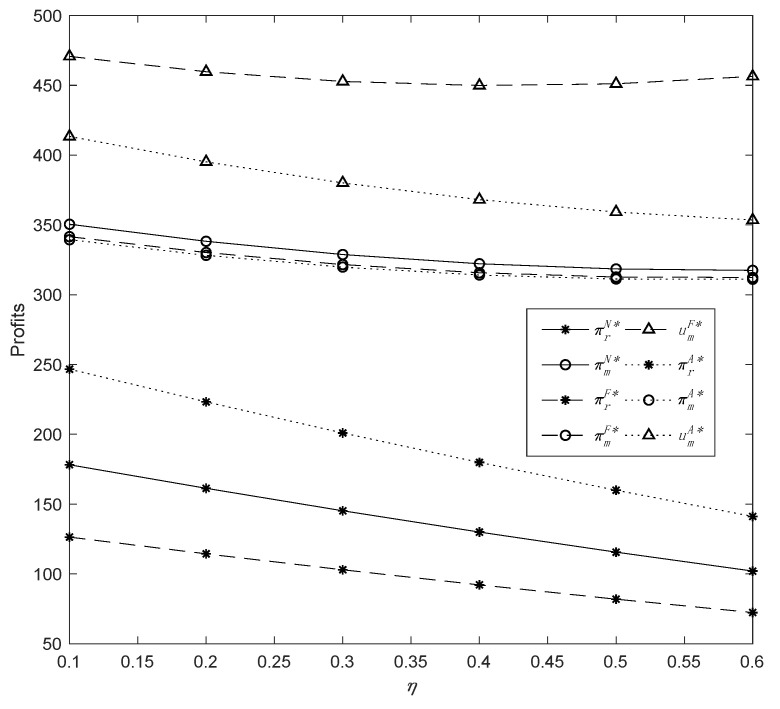
The change in profits with parameter *η*.

To begin, we explored how the CEA (*η*) affects demand, profits, and manufacturers’ utilities by setting *η* ϵ [0, 0.58], *δ* = 0.4, *λ* = 0.6, and *μ* = 0.3; we derived the results as presented in Figure 1 and Figure 2.

Figure 1 and Figure 2 show that CEA (*η*) plays an important role in determining optimal decisions. In all three models, the enhancement of CEA has a negative outcome on the demand for common products but a positive impact on that for green ones. When the demand for common products and green products are equal under Model *F*, we can obtain a threshold value of CEA, that is, η=0.42. Thus, when the manufacturer is fairness-concerned, and *η* is around 0.42, the demand for green products will be greater than the demand for common products; when the manufacturer has an altruistic preference, the demand for common products is always greater than that for green products. This indicates that *η* has a more significant impact on product demands when the manufacturer has fairness concern than when it has an altruistic preference. Meanwhile, the manufacturer’s profit from different models reveals a general trend of slight decline. In particular, the manufacturer’s profits in Models *F* and *A* are basically identical. This suggests that since the manufacturer is the market leader and owns two sales channels, the manufacturer loses slightly more profit in the offline channel than it gains additional profit in the direct channel in Model *F*, and vice versa in Model *A*; therefore, the comprehensive influence of CEA on the manufacturer is quite limited. In addition, *η* has a roughly steady effect on the utility of the manufacturer’s fairness concern but a negative effect on the utility of altruistic preference.

### 5.2. Impact of Consumer’s Channel Preference δ

**Figure 3:**
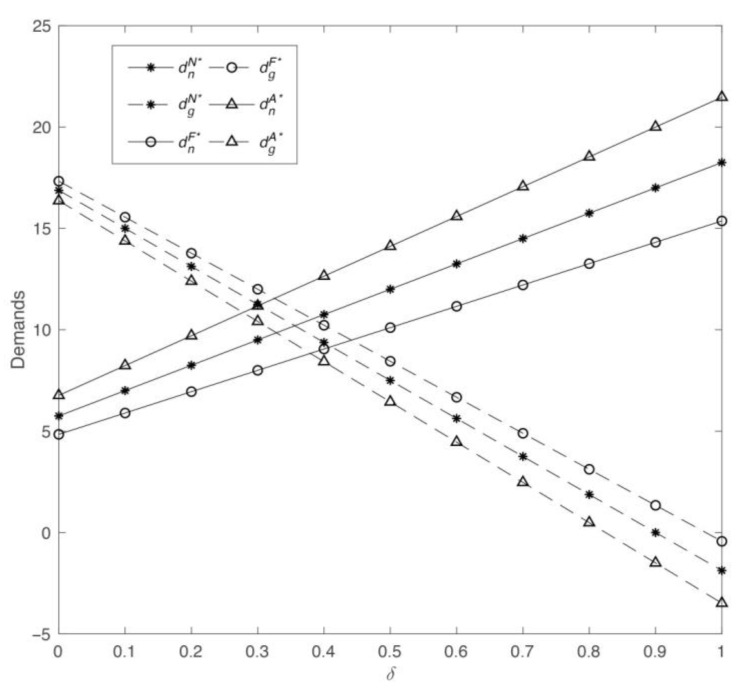
The change in demands with parameter *δ*.

**Figure 4:**
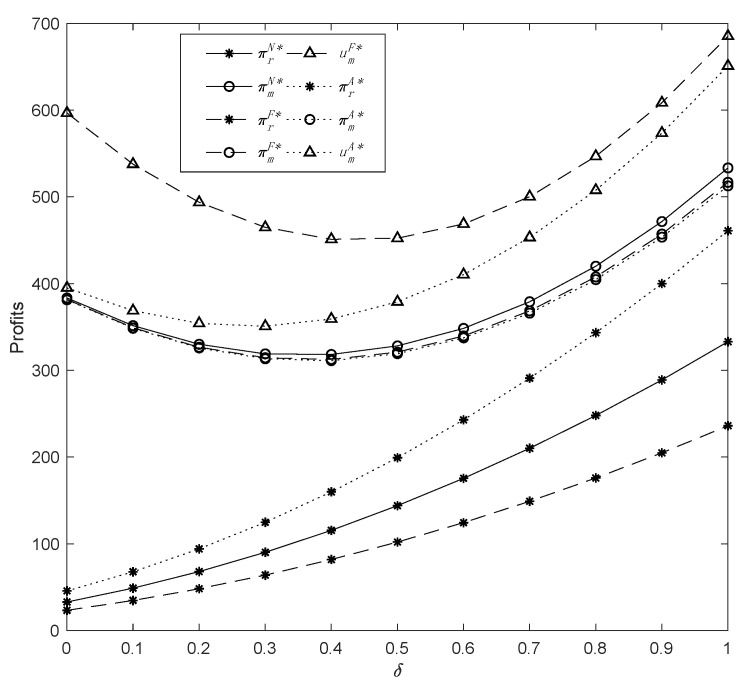
The change in profits with parameter *δ*.

Subsequently, we investigate how the consumer’s channel preference *δ* influences demand, profits, and manufacturer’s utilities within the three models. Setting *η* = 0.5, *λ* = 0.6, and *μ* = 0.3, the effects of *δ* on the optimal outcomes are illustrated in Figure 3 and Figure 4.

Going by Figure 3, as consumer preference for the offline retail channel improves, the demand for common products increases, and the demand for green products drops. A threshold value of *δ* can be calculated when the demand for both common and green products is equal under Model *A*, which is 0.27. Likewise, the *δ* threshold value of Model *F* can be derived as 0.42. Therefore, we can observe that when *δ* is less than or equal to 0.27, the market for green products is superior to that of common products, and when *δ* is greater than 0.42 or so, consumers prefer common products regardless of the manufacturer’s fairness preferences. Figure 4 on its part illustrates that, the retailer’s profit increases considerably with the growth in *δ*. It is apparent that *δ* has a more significant positive outcome under Model *A* and a slightly lighter impact under Model *F*, whereas Model *N* is somewhere between the two scenarios. Moreover, it is evident that the profits of the manufacturer under the three models exhibit comparable tendencies. Particularly, when *δ* is small, the manufacturer’s profit decreases with an increase in *δ*, whereas when *δ* is greater than 0.4, the manufacturer’s profit becomes positively correlated with *δ*. This means that when consumers increasingly prefer to purchase common commodities offline, namely, when *δ* is greater than 0.4, the increase in demand drives up wholesale and retail prices, thereby increasing the profits. This indicates that regardless of whether the manufacturer has fairness preferences, supply chain participants benefit from consumers’ increasing preference for the retail channel.

Moreover, in view of the manufacturer’s utilities, an increase in *δ* initially decreases utilities before subsequently increasing them. However, as opposed to the utility of fairness concern, the decreasing trend of altruistic preference utility is more gradual, whereas the increasing trend is more significant. This is because when consumers turn to online shopping, the demand for common products will fall sharply, leading to a reduction in utilities, especially when the manufacturer has a fairness concern behavior. Nevertheless, when consumers’ offline shopping intentions become stronger, the demand for common products increases significantly, thereby enhancing the manufacturer’s utility in terms of fairness concerns and altruistic preferences. Moreover, it can be seen from the comparison that when the values of *δ* are sufficiently large, the fairness concern utility begins to show an upward trend.

### 5.3. Impact of Fairness Concern λ

**Figure 5:**
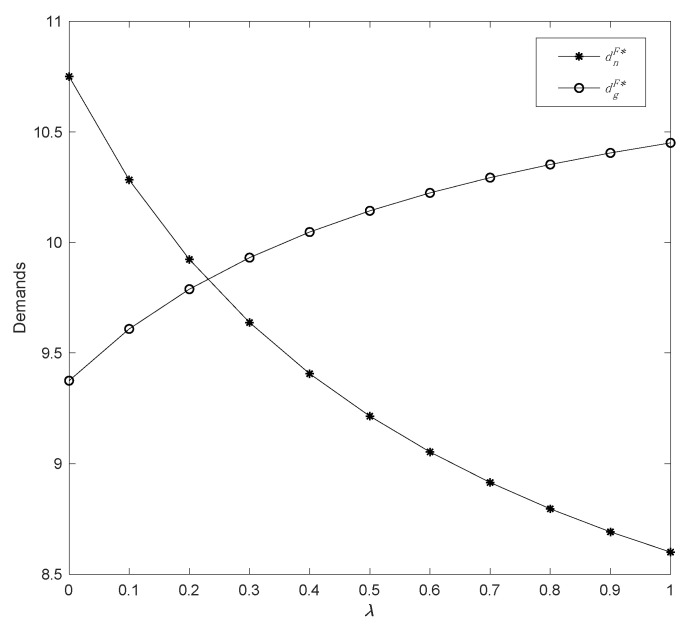
The change in demands with parameter *λ*.

**Figure 6:**
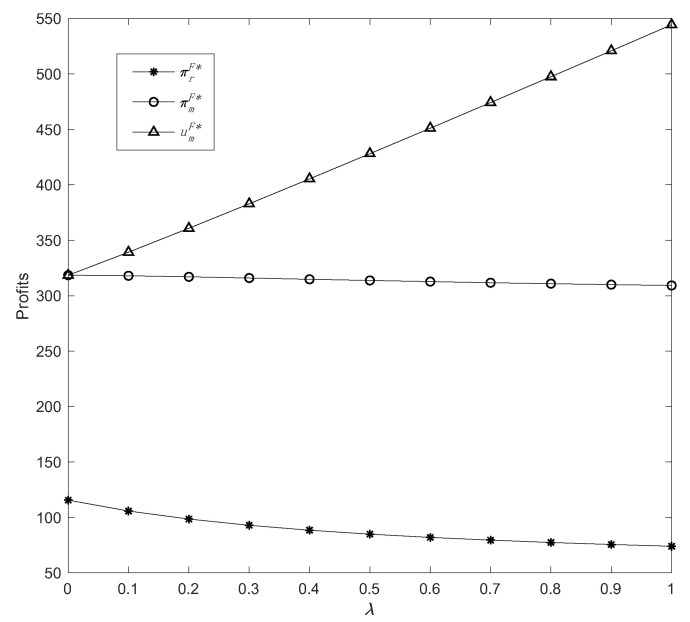
The change in profits with parameter *λ*.

Then, by varying the fairness concern coefficient in a range *λ* ϵ [0, 1], we can examine the effect of fairness concern on demand and profits. With the assumptions of *η* = 0.5 and *δ* = 0.4, we obtain the following results in Figure 5 and Figure 6.

Figure 5 illustrates that the demand for common products decreases as the fairness concern coefficient increases, whereas the demand for green products increases. Furthermore, the decline in common products is more dramatic than the increase in green products, which indicates that fairness concern behavior has an essential impact on common products. Moreover, in the condition where the demand for the two types of products is equivalent, the threshold value of *λ* can be obtained as 0.23. In other words, when *λ* < 0.23, common products have a larger share of market demand; however, when *λ* > 0.23, the sales of green products become better than the common ones. Consequently, the manufacturer’s fairness concern behavior with respect to the retailer results in an increase in the retail price of common products, leading to a considerable reduction in demand and, ultimately, a negative impact on common products. On the other hand, as the price of common products increases, a portion of potential consumers shift to green products, leading to a progressive increase in demand for green products.

As illustrated in Figure 6, manufacturer and retailer profits are adversely correlated with the degree of fairness as they steadily decrease with an increase in *λ*. This is because the retailer’s profit margins have reduced as a result of the decreased demand for common products. However, while some profits from the offline retail channel are lost, the manufacturer’s overall profits remain relatively stable because of the growing demand from the direct online channel. In contrast to its effect on profits, fairness concern behavior is beneficial to the manufacturer’s utility. That is to say, the higher the degree of the manufacturer’s fairness concern, the greater his utility. In sum, affected by the growing demand for green products via the direct sales channel, the manufacturer’s profit declines slightly but remains significantly higher than the retailer’s profit, implying that the manufacturer’s utility continues to rise in general.

### 5.4. Impact of Altruistic Preference μ

**Figure 7:**
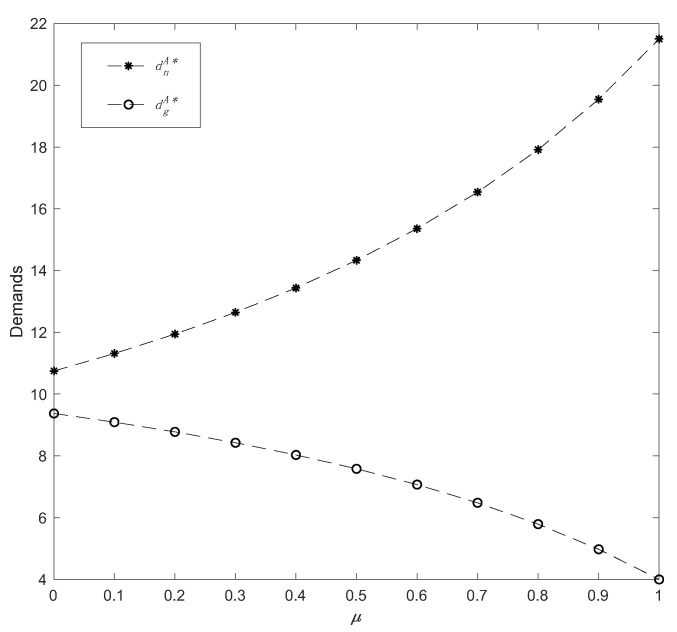
The change in demands with parameter *μ*.

**Figure 8:**
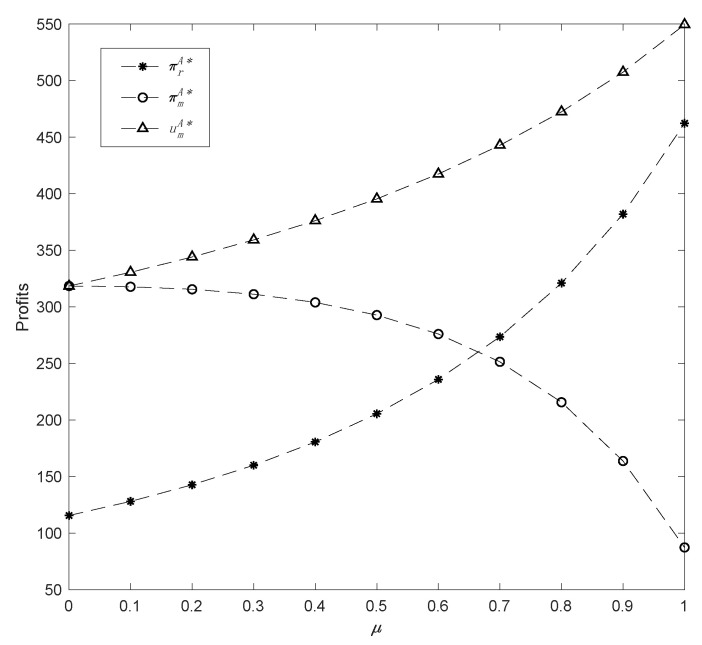
The change in profits with r parameter *μ*.

Finally, by varying the altruistic preference coefficient in a range *μ* ϵ [0, 1], the impacts of altruistic preference on demand and profits are described. Assuming *η* = 0.5 and *δ* = 0.4, the related outcomes are as shown in Figure 7 and Figure 8.

According to Figure 7, the manufacturer’s altruistic preference behaviors have opposite effects on the demands for common products and green products. Specifically, *μ* has a favorable effect on common products demand, but a detrimental effect on green products demand, and comparatively, the increase in demand for common products is more significant than the decline in green products. This shows that while altruistic behavior might stimulate the retail market to some extent, it has a negative impact on the market for green products and leads to a loss of some potential green consumers, which contradicts the concept of green thinking.

Furthermore, as can be seen in Figure 8, the greater the *μ*, the greater the influence on the profits of supply chain participants, especially the manufacturer, whose profit is negatively affected by an increasing *μ*, and the decline is more critical when the value of *μ* is larger. Conversely, the retailer’s profit is mushrooming continuously with the increase in *μ*, demonstrating that the retailer’s profit is rising as consumers demand more common products. However, although the manufacturer’s profit drops, the utility steadily increases as *μ* increases. In short, if the manufacturer increasingly focuses on altruistic preference, it will be advantageous for the retailer, but detrimental to its own profit. As a result of calculating the critical point where the profit of the manufacturer equals the profit of the retailer, it is possible to derive that *μ* is 0.67. That is to say, the manufacturer’s altruistic preference should not exceed 0.67; otherwise, the manufacturer’s profit will be lower than that of the retailer. It can be inferred by combining Figure 6 and Figure 8 that the whole green supply chain performance is highest when the manufacturer has an altruistic preference, accompanied by rational decision making, and lowest when the manufacturer is fairness-concerned, thereby indicating that altruistic preference is a positive social indicator. However, in practice, the degree of altruistic preference of the manufacturer is generally low.

### 5.5. Discussion

Our results show that CEA always has a positive effect on green products in all three models, implying that the stronger the CEA, the greater is the demand for green consumption. This is consistent with the partial findings presented by a variety of studies [35,45,47,49]. It is important to note that the influence of CEA on product demand is related to the fairness preferences of the manufacturer. Specifically, CEA has a more significant impact on demand when the manufacturer has fairness concerns. Likewise, consumer channel preference affects both prices and demand for green and common products, but to different degrees. It is enthralling to compare these results with those reported by Meng et al. (2021) [58], who found that consumers’ preference for offline channels was conducive to common products, but detrimental to green ones. Moreover, it is interesting to note that the retailer’s profit reduces as CEA becomes stronger and declines as consumers’ channel preference decreases, whereas the manufacturer’s profit in this case is almost unaffected by CEA. In addition, consumers’ preferences for different channels result in more dramatic changes in fairness concern utilities.

These outcomes partially contradict the findings of Zhang et al. (2019) [51], who discovered that CEA increases manufacturer and retailer profits. A possible explanation is that the supply chain structures are different. We explore a dual-channel supply chain that distributes common and green products, whereas Zhang et al. (2019) [51] analyzed a single-channel supply chain with one type of green product. Therefore, manufacturers with two sales channels are less affected by CEA, ensuring stable operations and competitiveness in the market. This implies that manufacturers’ selection of multiple channels to offer heterogeneous products has an apparent impact on the performance of green supply chains.

Additionally, if the manufacturer becomes increasingly prone to fairness concerns, the manufacturer and retailer both increase the wholesale and retail prices of common products to boost profits, resulting in a decline in demand for common products and an increase in demand for green products. At the same time however, although an increase in the coefficient of the manufacturer’s fairness concern is disadvantageous to both the retailer and manufacturer profits, the decrease in manufacturer profits is negligible because of the increased demand for green products. Therefore, we have additional evidence for the points revealed by Jian et al. (2021) [62] and Wang et al. (2020) [63], who stated that manufacturers’ fairness concerns indicated a loss of retailer and manufacturer profits. This demonstrates that a manufacturer’s fairness concern behavior is always detrimental to supply chain performance, especially for downstream retailers. There is another finding which slightly differs from the results of Jian et al. (2021) [62], who found that the prices of green products gradually increase with the degree of fairness concern. We rather find that the prices of green products remain unaffected by manufacturers’ fairness preferences, whereas demand grows with surging fairness concerns. This is also a remarkable explanation as to why manufacturers’ profits remain relatively stable.

Furthermore, altruistic preference generates completely opposite effects on common and green products. Specifically, as altruistic preference increases, demand for common products increases, whereas demand for green products drops. This is because manufacturers’ altruistic preference behaviors compel them to reduce wholesale and retail prices and transfer a portion of their profits to retailers to balance the performance. Hence, the retailer’s profit and the manufacturer’s utility are positively correlated with altruistic preference. These results are in partial agreement with those obtained by Huang et al. (2019) [69] and Ma et al. (2021) [72]. However, it is recognized that when manufacturers exhibit strong altruistic preferences, the effect on their profit is significantly negative. Further, this finding diverges from the outcomes of Ma et al. (2021) [72], who suggested that manufacturers can benefit from their altruistic preference behaviors. This discrepancy could be attributed to the differences in sales channels and products. It follows that there is uncertainty regarding the impact of manufacturers’ fairness concerns and altruistic preferences on decisions as well as on other members of the supply chain. It is necessary as a policymaker to assess the preferences of different supply chain members comprehensively before making a final decision regarding the future of the enterprise, which is dependent on the structure of the supply chain and the characteristics of the products sold. This will allow the enterprise to optimize its performance through a more flexible and feasible plan.

## 6. Conclusions

In the framework of the green economy and sustainable development, the market for green products is experiencing continuous growth as CEA steadily increases [80], and numerous companies have taken steps to achieve sustainable competitiveness by implementing green practices and providing environmentally friendly commodities [81,82]. Because policymakers are rarely completely rational, they tend to focus on profit distribution along the supply chain, particularly when the R&D of green products lies with manufacturers [83]. It is proposed in this work that a manufacturer provides green products with a direct selling channel to consumers, whereas the retailer sells common products via brick-and-mortar stores. Given the growing importance of fairness preference issues in the green market, the fairness concerns and altruistic preferences of the manufacturer have been incorporated into our models. In this study, we derive the optimal decisions by comparing and analyzing three different modeling scenarios. Moreover, the influences of changing fairness concerns, altruistic preferences, consumers’ channel preferences, and CEA on optimal prices, demands, profits, and utilities are generated and discussed, and we draw relevant conclusions.

### 6.1. Main Findings

There are several interesting findings that emerge from our study. First, as a retailer only carries common products, raising consumer awareness of environmental protection adversely affects their sales. As for the manufacturer, while producing green products incurs higher costs, they are able to compensate for this loss by selling substitute products. Thus, the increase in consumer awareness of environmental protection has a negligible impact on them. This is consistent with Khan and Qianli’s findings (2017) [84], who concluded that green production could reduce firm performance to a certain extent. The development of a green economy and the promotion of a green consumption system, however, have long-term benefits for society, the environment, and the economy. It is therefore imperative that retailers actively invest in and distribute green products, publicize the green economy, and encourage consumers to engage in green consumption. In addition to maintaining the production of substitutes, manufacturers should strive to reduce green costs, stimulate green technology innovation, mobilize downstream retailers to share green investments, and seek government subsidies to boost green production.

Second, one of the more notable findings to emerge from this study is that, when over 40% of consumers prefer to spend offline, they are more willing to purchase common products regardless of the manufacturer’s attitude toward fairness. In this case, as *δ* increases, both the retailer and manufacturer profits increase. This is because, in reality, whereas some consumers are willing to pay a premium for green products, there are still quite a few consumers who opt for affordable alternatives. Although this benefits both manufacturers and retailers, it is detrimental to the long-term development of a green and sustainable supply chain in the long run. Therefore, strengthening CEA is a critical issue for achieving sustainability goals.

Lastly, for manufacturers that possess online and offline channels and provide diverse products, their fairness preferences have little influence on their profits, but retailers are affected differently. The retailer’s profit is highest when the manufacturer has altruistic preferences, followed by a circumstance in which the manufacturer is unconcerned with fairness. Finally, the retailer’s profit is lowest when the manufacturer is fairness concerned. These results, though precursory, indicate that policymakers should mind the fairness concerns and altruistic preference behaviors of supply chain participants so as to design appropriate strategies that will enable them to survive changing competitive market trends and promote sustainable development in the social, economic, and environmental fields.

### 6.2. Implications for Theory

The study contributes theoretically to the field. This study aims to extend the pricing model to a dual-channel green supply chain that takes into account manufacturers’ differing fairness preferences and incorporates consumer awareness of environmental protection and channel preferences so that pricing decisions can be determined in various situations based on these preferences. The study incorporates both green and common products into the supply chain, demonstrating that manufacturers’ fairness concerns and altruistic preferences greatly affect final decisions and influence retailer profitability and supply chain performance differently. Furthermore, a comparison between the optimal solutions in three scenarios is presented in this paper, along with observations of the influence of CEA, consumer channel preferences, manufacturer fairness concerns, and altruistic preference coefficients on the optimal solution. The results show that CEA is conducive to the expansion of the green product market, although the effects of CEA and channel preferences on the supply chain are partly dependent on the manufacturer’s fairness preferences. This means that the diversity of manufacturers’ fairness preference behaviors complicates supply chain decisions. This paper discusses the possibility that implementing green practices and adopting alternative fairness preferences may lead to improvements in a greener and more efficient dual-channel supply chain and provide recommendations to enterprises regarding their environmental sustainability decisions. To summarize, this study provides theoretical guidance for strengthening pricing strategies under the influence of different behavioral preferences of supply chain members in order to allow enterprises and supply chains to make sustainable green development decisions.

### 6.3. Implications for Practice

This study provides the following practical implications and policy recommendations for green supply chain management:

(1) According to the previous analysis, when only manufacturers participate in green production and markets, the effect on the development of a green supply chain is limited. Therefore, it is critical to integrate retailers into green practices and provide green products offline. Manufacturers and retailers should design more attractive sales strategies for green products and strengthen consumers’ multi-channel purchasing concepts to ensure the stable operation of dual-channel supply chains, which contributes to the sustainability of green supply chains.

(2) Consumers play a vital role in green supply chains. Hence, boosting CEA levels is crucial in green product marketing. Supply chain parties can promote green practices through a variety of methods, such as advertising and education, to raise public awareness. This orchestrates a successful long-term solution for environmental, societal, and sustainability goals and also assists the government in accelerating the construction of green markets.

(3) Essentially, manufacturers always undertake green R&D and manufacturing. As green substances are substantially more costly than traditional ones, and firms do not receive considerable government subsidies, which constitute important determinants of manufacturers’ fairness concern behaviors, the consequences are detrimental to both manufacturers and retailers. Therefore, to alleviate the fairness concerns of manufacturers, the government can provide financial and technological incentives to prompt enterprises to engage in green production. Retailers on their part can also motivate manufacturers to invest in green development by sharing R&D related to green technologies, thereby generating a win-win situation.

(4) Retailers’ profits decline in response to manufacturers’ fairness concerns and increase in response to their altruistic preferences. This suggests that the impact of manufacturers’ fairness behaviors on retailer profits is not fixed. In sum, retailers should take into account the different behavioral preferences of manufacturers and assess the influence on their profits to arrive at more negotiable and logical conclusions. While manufacturers’ altruistic preferences are beneficial for retailers, they are not always favorable to the establishment of sustainable green supply chains. Hence, it is necessary for policymakers to adapt to market development rules and adopt greener and more sustainable strategies.

### 6.4. Limitations and Future Research

It is acknowledged in this paper that there are some limitations that require further research in order to be addressed. As in the majority of previous works, the model is simplified by involving a single party at each stage of the supply chain. It should be noted, however, that pure monopolies are rare in the real world, and each stage typically comprises multiple parties with competing relationships. As a result, future research may examine competitiveness among supply chain parties. Further, this study assumes that market information is symmetrical. However, in real markets, supply chain members typically hide their proper information. Thus, further studies could be conducted to construct asymmetric information models. Moreover, we only evaluated the manufacturer’s fairness preference behaviors but not that of the retailer. In fact, there is a possibility that supply chain participants have fairness concerns coexisting. Hence, it would be thrilling to integrate the fairness concern behaviors of other individuals and investigate their influence on outcomes. The study could further examine the coordination scheme of the supply chain and propose appropriate contracts to improve the performance of the supply chain. We conducted a static analysis regardless of the impacts of disturbance factors and time; however, future studies could consider determining green supply chain decisions more objectively from a dynamic perspective.

## Figures and Tables

**Table 1:** Comparisons between this paper and related literature.

Papers	Pricing Strategy	Dual-Channel Supply Chain	Fairness Concern	Altruistic Preference	CEA	Consumers’ Preference
Jian et al. [62]	√	√	√			
Wang et al. [63]	√	√ (E-commerce supply chain)	√		√	
Tian et al. [52]	√	√ (Multi-channel)				√
Zhang et al. [35]	√	√			√	
Wan et al. [68]	√	√		√	√ (Low-carbon preference)	
Huang et al. [69]	√			√	√	
Wang et al. [55]	√	√				√
Ma et al. [72]	√	√		√		√
Meng et al. [58]	√	√			√	√
Niu et al. [67]		√	√	√		
This paper	√	√	√	√	√	√

**Table 2:** Notations.

Notation	Description
$c_{n}$, $c_{g}$	Production cost per unit of the common green product and green product, $c_{g}>c_{n}$
$\omega_{n}$	Wholesale price per unit of the common product
$p_{n}$	Offline retail price of the common product, $p_{n}>\omega_{n}>c_{n}$
$p_{g}$	Online selling price of the green product, $p_{g}>c_{g}$
a	The base market potential
k	Consumer’s sensitivity coefficient to the retail price, 0<k<1
γ	Cross price elasticity of demand, k>γ>0
τ	Greenness level of the green product
η	Consumer environmental awareness (CEA), 0≤η≤1
δ	Consumer’s preference coefficient for the offline retail channel, 0≤δ≤1
β	Cost-coefficient of green degree
λ	Fairness concern coefficient, 0<λ<1
μ	Altruistic preference coefficient, 0<μ<1
$d_{n}$, $d_{g}$	Demands for the common and green product
$\pi_{m}$	Profit of manufacturer
$\pi_{r}$	Profit of retailers
$u_{m}$	Utility of manufacturer
N*, F*, A*	Optimal solutions for Models *N*, *F*, and *A*

Where the subscript *n* denotes the common products, *g* is the green products, *m* represents the manufacturer, and *r* is the offline retailer.

## Data Availability

Not applicable.

## Appendix A

### Appendix A.1

**Proof** **of** **Proposition** **1.** In this set-up, we solve the retailer’s profit function first. The first partial derivative of $\pi_{r}$ to $p_{n}$ can be shown as:$\;\partial \pi_{r}/\partial p_{n}=\delta a+\omega_{n}-2p_{n}+\gamma p_{g}-\tau \left(\eta -1\right)$, the second partial derivative of $\pi_{r}$ to $p_{n}$ is:$\;\partial^{2}\pi_{r}/\partial p_{n}^{2}=-2$, thus equating the first partial derivative to 0, we obtain $p_{n}=\left(\delta a+\omega_{n}+\gamma p_{g}+\tau -\tau \eta \right)/2$. Then, we substitute the $p_{n}$ into Equation (4) and we have the first partial conditions which are $\partial \pi_{m}/\partial \omega_{n}=\left(\delta a+c_{n}-\gamma c_{g}+2\gamma p_{g}+\tau -\eta \tau -2\omega_{n}\right)/2$, $\partial \pi_{m}/\partial p_{g}=\left(a\left(2+\delta \gamma -2\delta \right)-\gamma c_{n}-c_{g}\left(\gamma^{2}-2\right)+2\gamma^{2}p_{g}-4p_{g}+2\eta \tau +\gamma \tau -\eta \gamma \tau +2\gamma \omega_{n}\right)/2$. The second partial derivative $\partial^{2}\pi_{m}/\partial \omega_{n}^{2}=-1$, $\;\partial^{2}\pi_{m}/\partial p_{g}^{2}=\gamma^{2}-2$, $\;\partial^{2}\pi_{m}/\partial \omega_{n}\partial p_{g}=\gamma$, hence the Hessian matrix can be shown as $H=(\begin{array}{c} -1\\ \gamma \end{array}\begin{array}{c} \gamma \\ \gamma^{2}-2 \end{array})$, then we obtain $\left|H\right|=2-2\gamma^{2}$, since 0<γ<1 is assumed, so that the Hessian matrix is negative definite. Then, the $\pi_{m}$ is jointly concave in $\omega_{n}$ and $p_{g}$. By setting, $\partial \pi_{m}/\partial \omega_{n}=0$, $\partial \pi_{m}/\partial p_{g}=0$ the optimal wholesale price for common products $\omega_{n}^{N*}$ and the optimal online sale price for green products $p_{g}^{N*}$ can be solved. Replacing $\omega_{n}$ and $p_{g}$ with $\omega_{n}^{N*}$ and $p_{g}^{N*}$ into $p_{n}$ then $p_{n}^{N*}$ can be obtained. Substituting $\omega_{n}^{N*}$, $\;p_{g}^{N*}$ and $p_{n}^{N*}$ into Equations (1)–(4), we can derive $d_{n}^{N*}$, $d_{g}^{N*}$, $\pi_{r}^{N*}$ and $\pi_{m}^{N*}$. □

### Appendix A.2

**Proof** **of** **Proposition** **2.** (1) When the demand for green products is zero, then $d_{g}^{N*}\leq 0$, so that $d_{g}^{N*}=\left(a\left(2+\delta \gamma -2\delta \right)+c_{n}\gamma +c_{g}\left(\gamma^{2}-2\right)+2\eta \tau +\gamma \tau -\eta \gamma \tau \right)\leq 0$, that is $\eta \leq \frac{2a-2c_{g}-2a\delta +c_{n}\gamma +\delta a\gamma +c_{g}\gamma^{2}+\gamma \tau }{\left(\gamma -2\right)\tau }$. Because we assume that 0≤η≤1, so we can obtain 0≤η≤min{1,M}, where $M=\frac{2a-2c_{g}-2a\delta +c_{n}\gamma +\delta a\gamma +c_{g}\gamma^{2}+\gamma \tau }{\left(\gamma -2\right)\tau }$. (2) When green products and common products are in demand, assuming $0<d_{g}^{N*}<d_{n}^{N*}$, solve $d_{g}^{N*}>0$, and $\mathrm{obtain}\;\eta >\frac{2a-2c_{g}-2a\delta +c_{n}\gamma +a\delta \gamma +c_{g}\gamma^{2}+\gamma \tau }{\gamma \tau -2\tau }$. From $d_{g}^{N*}<d_{n}^{N*}$, we can obtain $\eta <\frac{2a+c_{n}-2c_{g}-3\delta a+c_{n}\gamma -c_{g}\gamma +\delta a\gamma +c_{g}\gamma^{2}-\tau +\gamma \tau }{\left(\gamma -3\right)\tau }$, and because 0≤η≤1, then we can derive max{0,M}≤η≤min{1,N}, where $N=\frac{2a+c_{n}-2c_{g}-3\delta a+c_{n}\gamma -c_{g}\gamma +\delta a\gamma +c_{g}\gamma^{2}-\tau +\gamma \tau }{\left(\gamma -3\right)\tau }$. □

### Appendix A.3

**Proof** **of** **Corollary** **1.** Based on the equilibrium solutions obtained in Proposition 1, we have $\frac{\partial \omega_{n}^{N*}}{\partial \eta }=-\frac{\tau }{2+2\gamma }<0$, $\;\frac{\partial p_{g}^{N*}}{\partial \eta }=\frac{\tau }{2+2\gamma }>0$, $\;\frac{\partial p_{n}^{N*}}{\partial \eta }=-\frac{\left(3+\gamma \right)\tau }{4\left(1+\gamma \right)}<0$, $\;\frac{\partial d_{n}^{N*}}{\partial \eta }=-\frac{\tau }{4}<0$, $\;\frac{\partial d_{g}^{N*}}{\partial \eta }=\frac{\tau \left(2-\gamma \right)}{4}>0$, $\;\frac{\partial \pi_{r}^{N*}}{\partial \eta }=-\frac{1}{8}\tau \left(\delta a-c_{n}+c_{g}\gamma +\tau -\eta \tau \right)$, and because $p_{n}>w_{n}$, so that $p_{n}-w_{n}=\delta a-c_{n}+c_{g}\gamma +\tau -\eta \tau >0$, then we obtain $\frac{\partial \pi_{r}^{N*}}{\partial \eta }<0$; $\frac{\partial \pi_{m}^{N*}}{\partial \eta }=\frac{\tau \left(a\left(2+\delta \gamma -3\delta \right)+\left(c_{n}+c_{g}\left(\gamma -2\right)\right)\left(1+\gamma \right)+\tau \left(\gamma -1-\gamma \eta +3\eta \right)\right)}{4\left(1+\gamma \right)}$, based on the assumption that $d_{n}>d_{g}$, thus $a\left(2+\delta \gamma -3\delta \right)+\left(c_{n}+c_{g}\left(\gamma -2\right)\right)\left(1+\gamma \right)+\tau \left(\gamma -1-\gamma \eta +3\eta \right)>0$, then $\frac{\partial \pi_{m}^{N*}}{\partial \eta }>0$ is obtained. □

### Appendix A.4

**Proof** **of** **Corollary** **2.** We omit the proof here since it is similar to the proof in Corollary 1. □

### Appendix A.5

**Proof** **of** **Proposition** **3.** Similar to the proof of Proposition 1, the reverse induction is applied here as well. In this set-up, we first obtain $p_{n}$ which can be represented as $p_{n}=\left(\delta a+\omega_{n}+\gamma p_{g}+\tau -\tau \eta \right)/2$. Then, we substitute the $p_{n}$ into Equation(5) and we have the first partial conditions which are $\partial u_{m}/\partial \omega_{n}=\left(\delta a+c_{n}-c_{g}\gamma +2\gamma p_{g}+\tau -\eta \tau -2\omega_{n}+\lambda \left(2\delta a+c_{n}-2c_{g}\gamma +3\gamma p_{g}+2\tau -2\eta \tau -3\omega_{n}\right)\right)/2$, $\partial u_{m}/\partial p_{g}=\left(2\eta \tau -c_{n}\gamma -4p_{g}+\gamma \left(2\gamma p_{g}+\tau -\eta \tau +2\omega_{n}\right)+\left(p_{g}\left(\gamma^{2}-4\right)-c_{n}\gamma +2\eta \tau +3\gamma \omega_{n}\right)\lambda -c_{g}\left(\gamma^{2}-2\right)\left(1+\lambda \right)+a\left(\delta \left(\gamma -2-2\lambda \right)+2\left(1+\lambda \right)\right)\right)/4$, the second partial derivative $\partial^{2}u_{m}/\partial \omega_{n}^{2}=-3\gamma /2-1$, $\;\partial^{2}u_{m}/\partial p_{g}^{2}=\left(\gamma^{2}-2\right)\left(1+\lambda \right)-\gamma^{2}\lambda /2$, $\;\partial^{2}u_{m}/\partial \omega_{n}p_{g}=\gamma \lambda /2+\gamma \left(1+\lambda \right)$, hence the Hessian matrix can be shown as $H=(\begin{array}{c} -3\gamma /2-1\\ \gamma \lambda /2+\gamma \left(1+\lambda \right) \end{array}\begin{array}{c} \gamma \lambda /2+\gamma \left(1+\lambda \right)\\ \left(\gamma^{2}-2\right)\left(1+\lambda \right)-\gamma^{2}\lambda /2 \end{array})$, then we obtain $\left|H\right|=\left(1-\gamma^{2}\right)\left(2+5\lambda +3\lambda^{2}\right)$, because 0<γ<1 and 0<λ<1, so that the Hessian matrix is negative definite. Then, the $u_{m}$ is jointly concave in $\omega_{n}$ and $p_{g}$. By setting $\partial u_{m}/\partial \omega_{n}=0$ and $\partial u_{m}/\partial p_{g}=0$, the optimal wholesale price for common products $\omega_{n}^{F*}$ and the optimal online sale price for green products $p_{g}^{F*}$ can be solved. Replacing $\omega_{n}$ and $p_{g}$ with $\omega_{n}^{F*}$ and $p_{g}^{F*}$ into $p_{n}$ then $p_{n}^{F*}$ can be obtained. Substituting $\omega_{n}^{F*}$, $\;p_{g}^{F*}$ and $p_{n}^{F*}$ into Equations (1)–(6), we can derive $d_{n}^{F*}$, $d_{g}^{F*}$, $\pi_{r}^{F*}$, $\;\pi_{m}^{F*}$ and $u_{m}^{F*}$. □

### Appendix A.6

**Proofs** **of** **Corollary** **3–5.** We omit the proofs here since they are similar to the proof in Corollary 1. □

### Appendix A.7

**Proof** **of** **Proposition** **4.** The proof is similar to that of Proposition 3 and is thus omitted here. □

### Appendix A.8

**Proofs** **of** **Corollary** **6–8.** We omit the proofs here since they are similar to the proof in Corollary 1. □

### Appendix A.9

**Proof** **of** **Corollary** **9.** From the Proposition 1, Proposition 3 and Proposition 4, we have $\omega_{n}^{F*}-\omega_{n}^{N*}=\frac{\left(\delta a-c_{n}+c_{g}\gamma +\tau -\eta \tau \right)\lambda }{4+6\lambda }>0$ and $\omega_{n}^{A*}-\omega_{n}^{N*}=\frac{\left(\delta a-c_{n}+c_{g}\gamma +\tau -\eta \tau \right)\mu }{2\left(\mu -2\right)}<0$, so that $\omega_{n}^{F*}>\omega_{n}^{N*}>\omega_{n}^{A*}$; $\;p_{n}^{F*}-p_{n}^{N*}=\frac{\left(\delta a-c_{n}+c_{g}\gamma +\tau -\eta \tau \right)\lambda }{4\left(2+3\lambda \right)}>0$ and $p_{n}^{A*}-p_{n}^{N*}=\frac{\left(\delta a-c_{n}+c_{g}\gamma +\tau -\eta \tau \right)\mu }{4\left(\mu -2\right)}<0$, so that $p_{n}^{F*}>p_{n}^{N*}>p_{n}^{A*}$; $\;d_{n}^{F*}-d_{n}^{N*}=-\frac{\left(\delta a-c_{n}+c_{g}\gamma +\tau -\eta \tau \right)\lambda }{4\left(2+3\lambda \right)}<0$ and $d_{n}^{A*}-d_{n}^{N*}=-\frac{\left(\delta a-c_{n}+c_{g}\gamma +\tau -\eta \tau \right)\mu }{4\left(\mu -2\right)}>0$, so that $d_{n}^{F*}<d_{n}^{N*}<d_{n}^{A*}$; $\;d_{g}^{F*}-d_{g}^{N*}=\frac{\gamma \left(\delta a-c_{n}+c_{g}\gamma +\tau -\eta \tau \right)\lambda }{4\left(2+3\lambda \right)}>0$ and $d_{g}^{A*}-d_{g}^{N*}=\frac{\gamma \left(\delta a-c_{n}+c_{g}\gamma +\tau -\eta \tau \right)\mu }{4\left(\mu -2\right)}<0$, so that $d_{g}^{F*}>d_{g}^{N*}>d_{g}^{A*}$; $\;\pi_{r}^{F*}-\pi_{r}^{N*}=-\frac{\left(\delta a-c_{n}+c_{g}\gamma +\tau -\eta \tau \right)\lambda \left(4+5\lambda \right)}{16{\left(2+3\lambda \right)}^{2}}<0$ and $\pi_{r}^{A*}-\pi_{r}^{N*}=-\frac{{\left(\delta a-c_{n}+c_{g}\gamma +\tau -\eta \tau \right)}^{2}\mu \left(\mu -4\right)}{16{\left(\mu -2\right)}^{2}}>0$, so that $\pi_{r}^{F*}<\pi_{r}^{N*}<\pi_{r}^{A*}$; $\;\pi_{m}^{F*}-\pi_{m}^{N*}=-\frac{{\left(\delta a-c_{n}+c_{g}\gamma +\tau -\eta \tau \right)}^{2}\lambda^{2}}{8{\left(2+3\lambda \right)}^{2}}<0$, then $\pi_{m}^{F*}<\pi_{m}^{N*}$, $\;\pi_{m}^{A*}-\pi_{m}^{N*}=-\frac{{\left(\delta a-c_{n}+c_{g}\gamma +\tau -\eta \tau \right)}^{2}\mu^{2}}{8{\left(\mu -2\right)}^{2}}<0$, then $\pi_{m}^{A*}<\pi_{m}^{N*}$. □
